# Linking hearing loss with noise exposure at road traffics in India

**DOI:** 10.6026/9732063002001211

**Published:** 2024-10-31

**Authors:** Barkha Yadav, Abhay D. Havle

**Affiliations:** 1 Department of ENT, Krishna Institute of Medical Sciences, Karad - 415110, Maharashtra, India; 2 Department of Otorhinolaryngology, Krishna Institute of Medical Sciences, Karad - 415 539, Maharashtra, India

**Keywords:** Noise-induced hearing loss (NIHL), age-related, right and left ear, noise exposure, link

## Abstract

Noise-induced hearing loss (NIHL) is the 2nd leading cause of loss of hearing. Therefore, it is of interest to evaluate the link between hearing loss and noise exposure due to traffic. 80 patients were divided into 2 groups (i.e group A and group B) 40 each and assessed for noise exposure with the help of Lutron SL 4033SD and loss of hearing with the help of WHO recommended guidelines. We found that, both right and left ear were affected. We come to conclude that, using personal protective equipment like earplugs and earmuffs is a simple and effective way to mitigate this health risk.

## Background:

According to Koh *et al.* [1] and Uimonen *et al.* [2] traffic noise is related with a number of short-term and long-term consequences. Because it interferes with your natural sleep pattern, you could discover that you are unable to fall or stay asleep. The activity of endocrine glands may be increased, high BP can be caused, heart rates can be affected, and blood composition can be altered, among other undesirable physiological and psychological impacts [3]. According to past studies also concluded that, it causes loss of hearing, mental illnesses, and discomfort [4, 5]. NIHL is the result of frequent and prolonged exposure to very loud noises [6, 7]. In urban populations, there is a preventable and predictable illness with a significant frequency from an epidemiological perspective. The audiogram reveals a similar sensori-neural hearing loss, with the most pronounced hearing impairment seen in the 4,000-Hz range, often referred to as the 4,000-Hz dip [1]. Occupational Safety and Health Association (OSHA) has created a time-weighted average (TWA) of ninety decibels to determine level of noise pollution [8]. When individuals are exposed to sound levels of approximately 85dB, they may experience a temporary decrease in their hearing ability, which is referred to as temporary threshold shift. This condition typically clears up within 24 hours after being exposed. With repeated and prolonged exposure, the threshold shift can become permanent [9]. Therefore, it is of interest to link hearing loss with prolonged exposure to noise in traffic.

## Materials and Method:

The current comparative cross-sectional study was conducted in the E.N.T department, KVV Karad starting from September 2022 to December 2023 in total of 18 months with total of 80 patients included from 5 different locations across the city. The environmental sound intensity levels were initially assessed using a digital sound level (SL) meter (Lutron SL 4033SD), capable of measuring from 30 to 130 dB with a resolution of 0.1 dB and an accuracy of ±1.5. These levels were recorded 3 times daily at each location starting from 800 to 1000 hours in the morning, 1300 to 1500 hours in the afternoon and 1700 to 1900 hours in the evening. Each reading lasted 10 minutes per point. Measurements were taken at sites where traffic personnel were stationed to assess their exposure to noise levels. The data from the SL 36 meter provided "dose" readout. The environmental software CD version SW-U801-WIN, supplied with the Lutron SL 4033SD, converted these dosimeter readings into TWA noise exposure levels as shown below.

Lden=10.log10 (1/24 [12. 10 L day/10 + 4. 10 L evening +5/10 + 8.10 L night +10/10])

Audiometric testing of the subjects was conducted 8 hours after the last noise exposure to remove the possibility of a temporary threshold shift. Hearing assessment was carried out amongst 40 police personnel exposed to traffic noises at busy chowk/intersection with otoscopic examination of the tympanic membrane and tuning fork test of each respondent was done. Similarly, 40 police personnel doing a desk job with less exposure to traffic noises (below 70 dB) were taken in another group and will be subjected to the same tests as mentioned above. Finally, PTA tests were done by clinical audiometer in a sound treated room (Model-ELKON 3 and 3 MULTI).

## Equipment:

Turning fork test (Frequency of 256,512 & 1024 Hz. test further included rinne test, weber test, schwabach test, absolute bone conduction (ABC) test and pure tone audiometry.

## Inclusion criteria:

[1] Patients age between 21 to 50 years.

[2] All genders

[3] Not more than 3 years of continuous exposure to traffic noise while on duty

[4] Willing to participate

## Exclusion criteria:

[1] Patients with pre-existing conductive or mixed hearing loss including CSOM or otosclerosis, previous significant medical history (*e.g:* Diabetes Mellitus, HTN, consumption of tobacco in any form. *etc.,*)

[2] Previous consumption of any ototoxic drugs (*e.g:* - AKT, Quinine, Diuretics *etc.*)

## Statistical analysis:

Categorical variables were assessed with Pearson's Chi-Square test for independence of attributes. Mean and standard deviation and comparison between group using Z test for 2 means (unpaired t test).

## Results:

Table 1 shows that, group-A had an average age of 35.47 years (SD = 7.39), while group-B had an average age of 33.28 years (SD = 9.41).Therefore, no statistically significant difference between the groups as the p value was 0.25. Table 2 shows that, group-A and those not exposed group-B, there was no significant difference in gender distribution between the groups as the p value was 0.35. Table 3 shows that, group A had 32.50% underweight (BMI<18.5), 37.50% normal weight (BMI 18.5 -23) and 30.00% overweight (BMI 23-27.5), with a mean BMI of 24.11±1.30. While group B had 35.00% underweight, 42.50% normal weight and 22.50% overweight with mean BMI of 23.20 ± 1.45. Therefore, there was no significant difference in BMI distribution between the groups (Chi-square = 0.59, p = 0.44). Table 4 shows that, in group A (exposed), 63.33% of individuals with NIHL were exposed for more than 3 years, compared to 36.67% exposed for 3 years. While in group B (not exposed), only 29.41% of individuals with NIHL were exposed for 3 years, while 70.59% were exposed for more than 3 years. Thus found notable difference. Table 5 shows that, group A showed higher proportion experienced hearing impairment with 55.00% had noise-induced hearing loss and 5.00% had sensori-neural hearing loss. Whereas, group B showed lower rates of hearing loss with 25.00% had noise-induced hearing loss and 7.5% had sensori-neural hearing. Thus, data showed statistically significant difference between the 2 groups.

Table 6 shows that, in group A, a total of 24 cases showed NIHL, out of which 14 i.e. 58.3%% had mild HL (26-40 dB), 6 i.e. 25% had moderate HL (41-60 dB) and 4 i.e. 16.6% had severe HL (61-80 dB). Thus, there is no profound hearing loss. Whereas, in group B a total of 13 cases showed SND, out of which 5 i.e. 38.4% had mild HL (26-40 dB), 3 i.e. 23.07% had moderate HL (41-60 dB) and 5 i.e. 38.4% % had severe HL (61-80 dB). Hence, there is no profound hearing loss. Moreover, a significant difference was seen among the 2 groups for hearing status & its extent indicating longer duration of exposure for severe HL to develop. Table 7 shows that, a notable disparity in the hearing threshold at frequencies of 2000 Hz and 4000 Hz as compared to other frequencies. This implies that the hearing damage of NIHL lies between 3000 to 6000 HZ & not characteristic at 4000 HZ. Table 8 showed that, a notable disparity in the hearing threshold at frequencies of 2000 Hz and 4000 Hz as compared to other frequencies. This implies that the hearing damage of NIHL lies between 3000 to 6000 Hz and is not characteristic at 4000 Hz. Thus found, no specific ear showed predominance in terms of NIHL in fact, both ears were affected showing bilateral involvement in almost all cases.

## Discussion:

In our study we found that, no difference for gender, age in years, and height in centimetres and weight in kg BMI in kg/m^2^ between groups. The noise level was from 49.9 dB to 129.3 dB and the TWA was 102.8dB to 133dB. Whereas in study by Thomas *et al.* [10] found, traffic noise levels ranged between 75 dB to 85 dB, occasionally 90 dB and 100 dB. Similar study conducted by Ingle ST *et al.* in Jalgaon, India, the noise levels observed at different points of the city ranged between 79.9 dB to 95.4 dB with TWA between 102.0 dB to 124.4 dB [11]. We also found in our study that patients with 40 years of age, who were exposed to the traffic noise for a period of more than 3 years, had more NIHL as compared to those exposed for a period up to 3 years. 24 out of 40 (60%) cases were having NIHL. Out of these 24 none had profound hearing loss however, in 14 (58.3%), 6 (2%) and 4 (16.6%) cases were having SND of mild (26-40 dB), moderate (41-55dB) and severe (61-80 dB) sensori-neural hearing loss respectively. These observations were in concordance with similar study of Gupta *et al.* on traffic personnel revealed that most of them had mild to moderate impairment [12]. The percentage of respondents with hearing loss stood at 60% in contrast to our study Win *et al.* [13] this percentage was 50.5% for those working for more than 15 years. In the present study testing in right and left ear was conducted separately using audiometry and showed no significant difference of predominance of hearing loss of either ear in study participants. This was in accordance with the study conducted by Karketta *et al.* on workers of chromite mines [14]. In our study, we also found significant difference in hearing threshold of right and left ear between the two groups. Jayesh *et al.* also showed an increased hearing threshold of 4000Hz in textile workers of Gujarat [15]. Another study in contrast to our study by "McBride *et al.* [16] and Francois-Xavier *et al.* [17] who showed notches at 4 kHz".

Traffic police working near high traffic are exposed to damaging noise for 8-12 hours a day. The typical equivalent continuous noise levels (Leq) in the workplace environment of traffic police are 87.5 dB (A), with the noise dose received in the range of 96-998.6 per day; a noise dose over 100 is deemed high. More crucially, hearing threshold alterations at low, mid and high binary frequencies for both ears were found to be significantly significant at the personal noise exposure level. It was also found that self-reported hearing loss utilizing screening questions and self-reported rating scales is a somewhat accurate estimate of hearing impairment as determined by audiometry [18]. One of the primary factors contributing to hearing loss is exposure to traffic noise. The following conclusions were reached: pressure horns should be illegal; public transportation should be favoured over private vehicles to reduce noise pollution; tree planting should be encouraged along urban highways wherever possible; and traffic cops should be encouraged to wear hearing protection devices like earmuffs or earplugs. Implementing intermittent breaks, such as lunchtime during the afternoon hours, may mitigate the continuous exposure of traffic policemen to noise. Additionally, rotating shifts weekly from high-noise intersections to quieter traffic points or junctions can further decrease the prolonged exposure to traffic noise experienced by these officers [19].

## Conclusion:

Data shows that very less speech frequencies are affected and patients may have few symptoms. Therefore, any level of noise-induced hearing loss (NIHL) can dull high-frequency sounds like whistles or buzzers. Thus, preventative measures should be taken to include regular health monitoring, implementing hearing conservation programs and enacting legislation.

## Figures and Tables

**Table 1:** Age range:

**Variables**	**Group-A (n=40)**	**Group-B (n=40)**	**Significance**
Age	35.47 ± 7.39	33.28 ± 9.41	p value=0.25*

**Table 2:** Gender distribution

**Gender**	**Group-A (n=40)**		**Group-B (n=40)**		**Significance**
	**No of cases**	**Percentage**	**No of cases**	**Percentage**	
Male	28	70.00%	30	75.00%	p value=0.35*
Female	12	30.00%	10	25.00%	

**Table 3:** BMI distribution

**BMI (Kg/m^2^)**	**Group-A (n=40)**		**Group-B (n=40)**		**Significance**
	**No of cases**	**Percentage**	**No of cases**	**Percentage**	
< 18.5 (underweight)	13	32.50%	14	35.00%	
18.5-23 (normal)	15	37.50%	17	42.50%	Chi square= 0.59
23-27.5 (Overweight)	12	30.00%	9	22.50%	p value= 0.44
Mean BMI	24.11 ± 1.30		23.20 ± 1.45		

**Table 4:** NHL with duration

**Duration of exposure (years)**	**Group-A (n=40)**		**Group-B (n=40)**		**Significance**
	**NIHL Present**	**NIHL Absent**	**NIHL Present**	**NIHL Absent**	
3 years	11 (36.67%)	6 (60.00%)	5 (29.41%)	21 (91.30%)	Chi square= 20.77
< 3 years	19 (63.33%)	4 (40.00%)	12 (70.59%)	2 (9.69%)	p value= 0.0001
Total	30 (100.00%)	10 (100.00%)	17 (100.00%)	23 (100.00%)	

**Table 5:** Hearing distribution

	**Group-A (n=40)**			**Group-B (n=40)**		**Significance**
	**No of cases**	%		**No of cases**	**%**	
Normal Hearing	16	40.00%	Normal Hearing	27	67.50%	Chi square- 7.514
SND (NIHL)	24	60.00%	SND	13	32.50%	p value- 0.23

**Table 6:** Hearing status

**Study Groups**	**Hearing status**					
	0-25 dB Normal	26-40 dB Mild HL	41-60 dB Moderate HL	61-80 dB Severe HL	> 81 dB Profound HL	
Group A	16	14	6	4	0	Chi square= 8.18
Group B	27	5	3	5	0	p value= 0.04

**Table 7:** Right ear

**Mean frequency**	**Right Ear**		
	**Group-A (n=40)**	**Group-B (n=40)**	**p-value**
250 Hz	22.0± 1.52	15.6± 1.34	>0.05
500 Hz	18.2± 1.46	8.20± 1.08	>0.05
1000 Hz	20.9± 2.0	10.22± 3.02	>0.05
2000 Hz	32.3± 2.50	9.14± 3.27	<0.0001
4000 Hz	37.6± 1.87	16.38± 3.25	<0.0001
8000 Hz	34.7± 2.55	21.3± 2.88	>0.05

**Table 8:** left ear

**Mean frequency**	**Left Ear**		
	**Group-A (n=40)**	**Group-B (n=40)**	**P-value**
250 Hz	22.8± 2.85	17.08± 1.29	>0.05
500 Hz	19.1± 1.89	12.18± 1.74	>0.05
1000 Hz	30.18± 2.0	10.43± 3.01	>0.05
2000 Hz	37.24± 2.5	12.31± 3.27	<0.0001
4000 Hz	36.2± 1.97	13.24± 3.27	<0.0001
8000 Hz	29.7± 1.47	18.10± 2.70	>0.05
